# The Potential of the Yeast *Debaryomyces hansenii* H525 to Degrade Biogenic Amines in Food

**DOI:** 10.3390/microorganisms3040839

**Published:** 2015-11-06

**Authors:** Mathias Bäumlisberger, Urs Moellecken, Helmut König, Harald Claus

**Affiliations:** Institute of Microbiology and Wine Research, Johannes Gutenberg University Mainz, Johann-Joachim-Becherweg 15, 55128 Mainz, Germany; E-Mails: m.baeumlisberger@gmx.net (M.B.); ursmoell@students.uni-mainz.de (U.M.); hkoenig@uni-mainz.de (H.K.)

**Keywords:** biogenic amines, yeasts, *Debaryomyces hansenii*, *Yarrowia lipolytica*, copper amine oxidase, cheese, grape must

## Abstract

Twenty-six yeasts from different genera were investigated for their ability to metabolize biogenic amines. About half of the yeast strains produced one or more different biogenic amines, but some strains of *Debaryomyces hansenii* and *Yarrowia lipolytica* were also able to degrade such compounds. The most effective strain *D. hanseniii* H525 metabolized a broad spectrum of biogenic amines by growing and resting cells. Degradation of biogenic amines by this yeast isolate could be attributed to a peroxisomal amine oxidase activity. Strain H525 may be useful as a starter culture to reduce biogenic amines in fermented food.

## 1. Introduction

**Figure 1:**
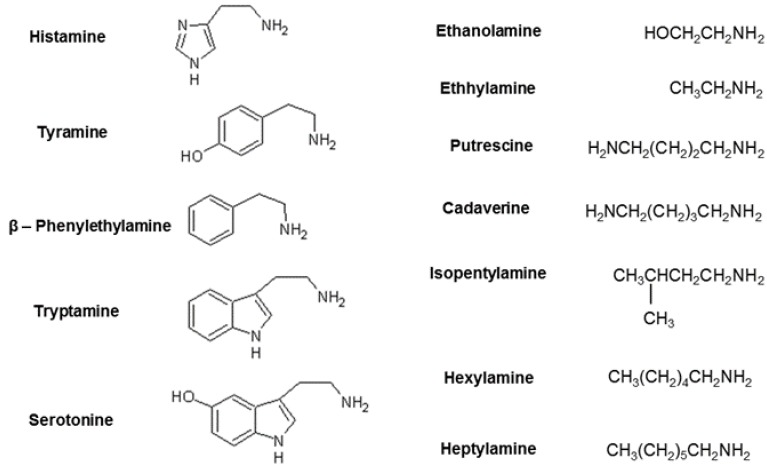
Biogenic amines. **left**: aromatic/heterocylic; **right**: aliphatic structures.

Biogenic amines (BA) are low molecular organic bases ubiquitously present in both pro- and eukaryotes. They are derivatives of ammonia bound to aliphatic, aromatic, or heterocyclic groups (Figure 1). According to the number of amino groups present in the structure, biogenic amines constitute three classes: monoamines (e.g., ethanolamine), diamines (e.g., cadaverine), and polyamines (e.g., spermidine).

BAs have important physiological regulatory functions in humans, especially as hormones and neurotransmitters (e.g., serotonine, adrenaline, noradrenaline). High concentrations can cause adverse health symptoms such as headaches, blood pressure crises, stomach cramps, and diarrhea [1]. These effects become enhanced by simultaneous consumption of alcohol, which inhibits the activities of detoxifying amine oxidases. In the worst case, synergistic effects may lead to anaphylactic shock [1,2,3].

**Figure 2:**
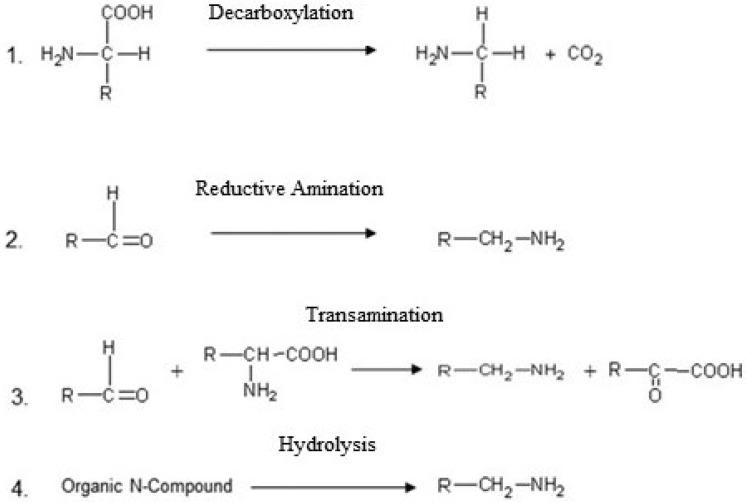
Formation of biogenic amines by 1. Enzymatic decarboxylation of amino acids, 2. Reductive amination of aldehydes and ketones, 3. Transamination reactions between aldehydes and amino acids and 4. Hydrolytic degradation of organic nitrogen compounds (e.g., phospholipids).

BAs in food are generally formed by microbial decarboxylation of amino acids, reductive amination of aldehydes and ketones, transamination or hydrolytic degradation of nitrogen compounds (Figure 2). The most common mechanism of formation is the microbial decarboxylation of amino acids, such as histidine to histamine. This type of reaction is catalyzed by amino acid decarboxylases. Some microorganisms use this reaction to generate an energy delivering proton gradient and/or to increase the cytoplasmic pH, protecting cells against acid damage [3].

BA concentrations between 1.0 and ≥100 mg/L (or mg/kg) have been detected in different foods [1]. Fish products contain the highest BA concentrations (mainly histamine) evolved by activities of enteric bacteria and of *Pseudomonas*. Tyramine, histamine, putrescine, and cadaverine are the most abundant BAs detected in cheese and fermented meat products associated with the activities of *Enterococcus*, *Lactobacillus*, *Staphylococcus*, and *Enterobacteriacea* [1]. Lactic acid bacteria of the genera *Lactobacillus*, *Leuconostoc*, *Pediococcus*, and *Oenococcus* are implicated in BA production in wine [4]. Strains of *Lactobacillus brevis* and *L. hilgardii* have been recognized as main producers of tyramine, histamine, and ethylamine in must [5]. Furthermore, the wine yeast *Saccharomyces cerevisiae* can produce significant amounts of ethanolamine and agmatine [6]. Non-*Saccharomyces* yeasts, like *Kloeckera apiculata*, *Candida stellata*, *Metschnikowia pulcherrima*, *Brettanomyces bruxellensis*, and the fungus *Botrytis cinerea* are additional producers of BA in grape must [4,6].

BA formation in food is traditionally controlled by limiting microbial growth through freezing, food additives, hydrostatic pressures, irradiation, and controlled packaging [7]. BA content in wine can be diminished by clean cellar practice, application of starter cultures [6,7,8], or bacteriolytic enzymes [9,10]. An emerging approach is the use of microorganisms to degrade BA in food [7]. Bacteria used as BA oxidizers include *Kocuria varians, Natrinema gari*, *Brevibacterium linen*, *Staphylococcus xylosus*, *Virgibacillus* and different species of *Lactobacillus* [7,11]. Callejon *et al.* [12] identified a laccase-like multicopper oxidase in *L. plantarum* J16 and *Pediococcus acidilactici* CECT 5930 strains, able to degrade BA in wine.

Thus, in addition to microbial cells, the application of amine oxidases have been suggested to degrade BA in food. Several studies and patents have already demonstrated the efficiency of amine oxidases, isolated from lactobacilli and fungi to remove BA in cheese, fish, beer, or wine [7,8]. Recently Callejon *et al.* [13] described a flavin-dependent oxidase from *K. varians* LTH 1540 which degraded putrescine and cadaverine even under harsh wine conditions (~pH 3.5; ≥10% *v/v* ethanol).

Surprisingly, only a few studies exist on the possible use of yeasts to control BA production, despite their importance as starters in fermentation of milk products, beer, and wine [14,15].

In this study we demonstrate that the yeast *Debaryomyces hansenii* H525 efficiently degrades a broad spectrum of BAs. This ability correlated with a peroxisomal amine oxidase activity.

## 2. Experimental Section

### 2.1. Yeast Strains, Identification, and Cultivation

The 26 yeast strains used are deposited at the local culture collection of the Institute of Microbiology and Wine Research, Johannes Gutenberg-University Mainz (Table 1). Species identification of *Debaryomyces hansenii* H 525 was confirmed by PCR amplification and sequencing of the internal transcribed spacer region (ITS) and 5.8S-rDNA. PCR conditions have been described by Sebastian *et al*. [11]. The primers used were ITS1 (Forward) TCCGTAGGTGAACCTGCGG and ITS4 (Reverse) TCCTCCGCTTATTGATATGC; the annealing temperature was 55 °C. The resulting PCR fragments were purified with the PCR Purification Kit (Quiagen, Germany). Sequencing was carried out by Eurofins MWG Operon (Germany). The partial 585 bp sequence showed highest identity (99%) with several *D. hansenii* strains. The yeasts were maintained on YEP medium (yeast extract 10 g/L, meat peptone 20 g/L, glucose 20 g/L). For preparation of cell-free extracts the yeasts were precultured in potato extract glucose broth (Roth, Germany).

### 2.2. Production of BA by yeasts

Production of BA by yeasts was monitored in a commercial direct-pressed red grape juice. The juice was heated in saturated steam for 20 min and inoculated at a relation of 1:10 with a preculture in YEP and incubated at 30 °C for up to 30 days without shaking. After centrifugation (16,000× *g*) the supernatant was analyzed for biogenic amines by thin-layer chromatography or HPLC (see below).

### 2.3. Degradation Experiments

Degradation of BA by yeast was tested with growing cells and stationary cell suspensions. The culture medium was a steam-treated direct pressed red grape juice with and without additions of 0.25 mM biogenic amines (tyramine, histamine, ethanolamine, phenylethylamine; Sigma, Munich, Germany). A different set of experiments contained additionally 0.25 mM serotonine, putresceine, isoamylamine, tryptamine, cadaverine, and hexylamine, as well separately or as a mixture. This grape juice was inoculated at a volume relation of 1:10 with a preculture in YEP and incubated while shaking at 30 °C for up to 10 days. The grape juice controls remained non-inoculated.

Further investigations were performed in a buffer system according to Leuschner *et al.* [11]. For this purpose, precultivated yeast cells were separated by centrifugation (3000× *g*) from the medium. The supernatant was discarded and the cells washed in 0.03 M sterile phosphate buffer (NaH_2_PO_4_ 13.8 g/L, Na_2_HPO_4_ 26.8 g/L; pH 7.0). After a further centrifugation step the cell pellet was resuspended in phosphate buffer with 0.25 mM of biogenic amines (tyramine, histamine, ethanolamine, phenylethylamine; Sigma, Munich, Germany) to obtain an optical density of 5.0 at 600 nm. Twenty mL of this cell suspension were transferred into sterile 100 mL Erlenmeyer flasks and incubated with shaking at 30 °C.

### 2.4. Analysis of Biogenic Amines

The qualitative analysis of BA in grape juice and phosphate buffer was done by thin layer chromatography (TLC) as described by Sebastian *et al.* [5]. BA were detected under UV-light (312 nm) after derivatization with dansylchloride (5-(dimethylamino)-naphtalene-1-sulfonchloride). For quantitative determinations, high-pressure liquid chromatography (HPLC) was conducted according to Christ *et al*. [16].

### 2.5. Determination of Amino Oxidase Activities in Cell-Free Extracts

#### 2.5.1. Preparation of Rough Cell Extract

*D. hansenii* H 525 was cultured in potato extract glucose broth (1.5 L) for 48 h at 30 °C under shaking (70 rpm). Cells were harvested by centrifugation (3000× *g*) and washed three times in 30 mM sodium phosphate buffer (pH 7.0). The resulting cell pellet was resuspended to 0.2 g/mL in the same buffer (120 mL). The cells were disrupted using a French^®^ pressure cell press (Aminco, American Instrument Company, Silver Springs, MD, USA) at a pressure of 20,000 psi in amounts of 30–40 per mL per cycle. The procedure was repeated three times for each sample. After disruption, protease inhibitor plus^®^ (Roth, Karlsruhe, Germany) was added (10 µl/mL) and samples were kept on ice all the time.

#### 2.5.2. Preparation of the Peroxisomal Fraction

The rough cell extract was centrifuged for 10 min at 1000× *g*, emerging supernatant S1 and pellet (P1). The latter was washed once more in the same volume of sodium phosphate buffer (30 mM, pH 7.0) and centrifuged for 10 min at 1000× *g*. P1 was discarded and the supernatant was combined with S1, which was centrifuged at 20,000× *g* to obtain P2 and supernatant S2.

#### 2.5.3. Extraction of Enzymes with Amino Oxidase Activities

S2 was discarded and P2 suspended in Triton X-100 (Roth, Karlsruhe, Germany; 1.0% in 30 mM sodium phosphate buffer, pH 7.0). The suspension was incubated for 24 h at 4 °C before centrifugation at 200,000× g (60 min). The resulting supernatant S3 was concentrated twice using Vivaspin 20™ spin columns (5000 MWCO, Sartorius, Göttingen, Germany) and stored until use at −20 °C.

The rough cell extract and S3 were tested for amino oxidase activity by incubation of 100 μL extract with the same volume of 1 mM solutions of different biogenic amines (tyramine, histamine, ethanolamine, phenylethylamine) for 24 h at 30 °C. The control contained only the biogenic amines in 30 mM sodium phosphate buffer (pH 7.0). Degradation of biogenic amines was checked by TLC as described above.

#### 2.5.4. Preparative Isoelectric Focusing (pIEF)

Concentrated S3 was dialysed for 48 h against deionized water. Three mL of this concentrate were mixed with 60 μL of 40% (*w/w*) ampholyte solution (Roti^®^Lyte, pH 3–5, Roth, Karlsruhe, Germany) and separated in the MicroRotofor™ Cell from BioRad (Munich, Germany) at 10 °C for 3 to 4 h at 1 W (cathode solution 0.25 HEPES, anode solution 0.5 M H_3_PO_4_). The 10 fractions obtained were analyzed by SDS-PAGE and degradation of biogenic amines monitored by TLC.

### 2.6. Determination of the Protein Content

Protein concentrations of the samples were determined with a Bradford based assay kit (Roti^®^-Quant, Karlsruhe, Germany).

### 2.7. Electrophoretic Procedures

Conditions for SDS-Polyacrylamide-gel-electrophoresis (SDS-PAGE) have been described recently [9,10]. Proteins were visualized by Coomassie staining or with the Pierce Silver Stain Kit™ (Thermo Scientific, Germany). The PageRuler™ Plus prestained protein ladder (Thermo Scientific, Heidelberg, Germany) was used as a molecular mass standard. Analytic isoelectric focusing (aIEF) and subsequent protein staining was carried out in Servalyt Precotes 3-10, following the recommendations of the manufacturer (Serva, Heidelberg, Germany). IEF gels were stained with Serva Violet 17 (Serva, Germany) or by using the silver-staining kit for the visualization of proteins.

### 2.8. Amplification of an Amino Oxidase Gene (CAO) from D. hansenii H525

Based on known gene sequences of yeast amino oxidases in public data bases (XM_461794, XM_011274212, XM_006684543, HE681719), primers were constructed for PCR amplification of a possible amino oxidase gene in *D. hansenii* H525: Aox2_Forward: TGGATATTGGTGAATATGGTGCT and Aox3_Reverse: GGAAGTCTTCAGGTGCTGGA. PCR conditions were the same as described above, but the annealing temperature was set at 60 °C. A 347 bp sequence was obtained and translated into the amino acids sequence (http://web.expasy.org/translate/).

## 3. Results and Discussions

### 3.1. BA Production by Yeasts

Twenty-six non-*Saccharomyces* yeast strains from different genera were screened for their ability to produce BA in grape juice (Table 1).

TLC analysis allowed separation and visualization of eight different BA. Five were detected in grape juice inoculated with the yeasts. About the half of 26 strains produced one or more different BA. Tyramine was generated by nine strains, putrescine, ethylamine, and phenylethylamine by five strains and histamine by a single strain of *D. hansenii* H199. Serotonine and isoamylamine were not found. As evident for *B. bruxellensis* and *D. hansenii*, BA production seems not to be a species-, but a strain-specific trait.

Several studies indicate that BA production by wine yeasts is a negligible phenomenon, the concentration of most amines being at non-detectable or very low levels [17]. According to Landete *et al*. [18], none of the studied yeast strains was able to produce at least one of the assayed amines (histamine, tyramine, 2-phenethylamine, putrescine, cadaverine, and tryptamine), in both synthetic medium and grape must.

Our experiments are in line with the study of Caruso *et al*. [6] who described several strains of wine yeasts to produce 2-phenethylamine and agmatine, at concentrations variable within each species from non-detectable to more than 10 mg/L. The contradictory data on biogenic amine producing capability of wine yeasts is a possible consequence of a high strain dependent variability of this metabolic feature.

**Table 1:** Production of BA in grape juice by the yeast strains investigated.

Species	Strain/Origin	BA Produced in Grape Juice *				
		Put	His	Tyr	Eth	Phe
*Dekkera bruxellensis*	H 600/Red wine	+	-	-	-	+
	H 604/Red wine	+	-	-	-	-
	H 608/Red wine	-	-	+	-	-
	H 612/Red wine	-	-	+	-	-
	H 614/Red wine	-	-	+	+	+
	H 615/Red wine	-	-	-	-	-
	H 623/Red wine	-	-	-	-	-
	H 628/Red wine	-	-	-	-	-
	H 629/Red wine	+	-	-	-	-
	H 631/Red wine	-	-	+	+	+
	H 635/Red wine	-	-	-	-	-
	H 637/Red wine	-	-	-	-	+
	H 644/Red wine	-	-	-	-	-
	H 645/Red wine	-	-	-	-	-
	H 647/Red wine	-	-	-	-	+
*Debaryomyces hansenii*	H 1/2-72/Garden Soil	+	-	+	-	-
	H 199/unknown	-	+	+	-	-
	H 524/Garden Soil	-	-	-	-	-
	H 525/Garden Soil	-	-	-	-	-
*Hanseniospora uvarum*	H 474/Apple	-	-	-	-	-
*Pichia membranaefaciens*	H 227/Wine	-	-	+	-	-
*Rhodotorula mucilaginosa*	H 365/unknown	-	-	-	-	-
*Schizosaccharomyces pombe*	H 81/Wine	+	-	+	+	-
	H 337/Wine	-	-	-	+	-
*Yarrowia lipolytica*	H 446/Isolate from beverage filter	-	-	-	-	-
*Zygosaccharomyces rouxi*	H 68/Wine	-	-	+	+	-

* Analyzed by TLC after 30 days cultivation in red grape juice; + detected; - not detected; Put: Putrescine; His: Histamine; Tyr: Tyramine; Eth: Ethylamine; Phenylethylamine.

### 3.2. Degradation of Biogenic Amines by D. hansenii H525

**Figure 3:**
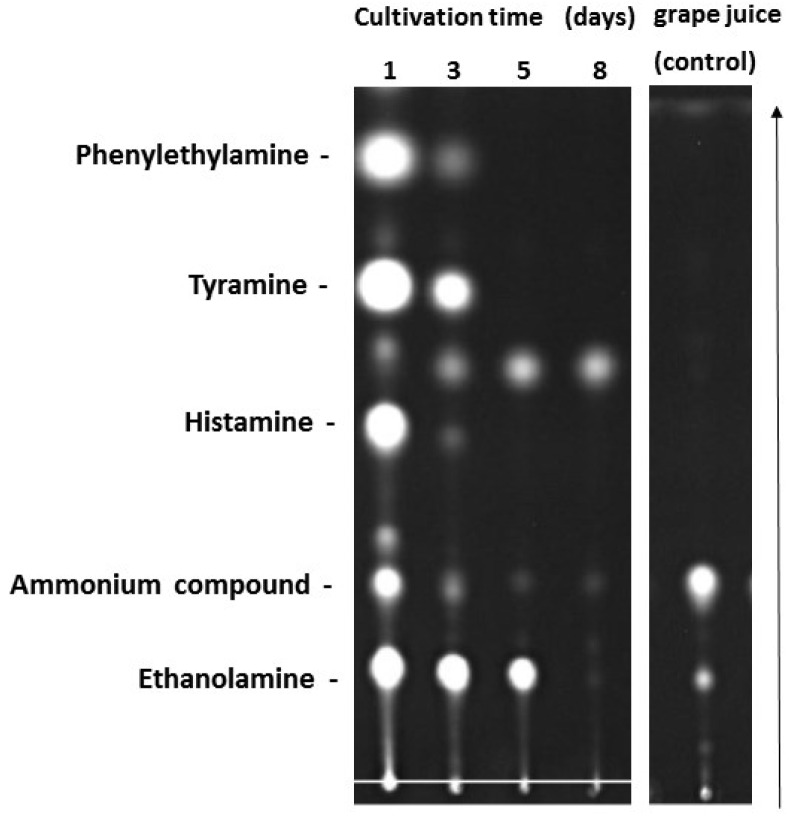
Kinetics of BA degradation by *D. hansenii* H525 as detected by TLC. Cultivation in red grape juice with 0.25 mM of each amine. The spots in the non-treated grape juice correspond to an intrinsic ammonium compound and to ethanolamine. The arrow indicates direction of separation.

We observed that the BA non-producer strains *Yarrowia lipolytica* H446 and *Debaryomyces hansenii* H525 obviously degraded ethanolamine, which was already present in the non-treated red grape juice control (Figure 3). When *D. hansenii* H525 was cultivated in red grape juice, all four added biogenic amines were completely degraded after eight days (Figure 3). These results were confirmed by sensitive HPLC measurements (detection limit~10 ng/mL). By the same method, we found that also *Y. lipolytica* H446 completely degraded the four biogenic amines after eight days of growth in grape juice (not shown). In addition to ethanolamine an unidentified ammonium compound was already present in the grape juice control. During yeast growth, these compounds vanished and were probably used as nitrogen sources. The presence of biogenic amines in grape juice had no significant influence on the total cell numbers of *D. hansenii* H525, which reached a maximum of 8 × 10^8^ cells/mL after eight days.

**Figure 4:**
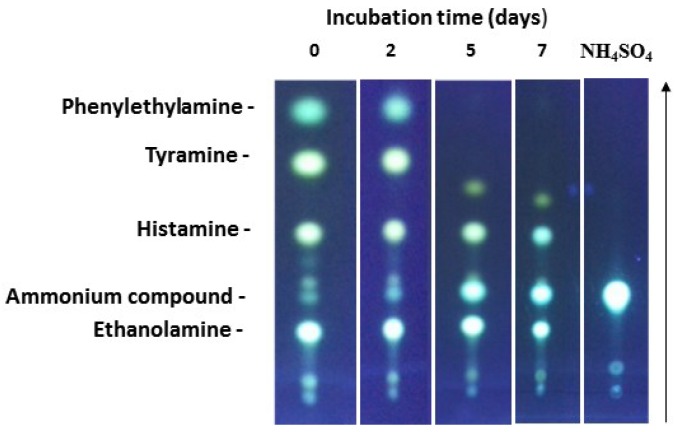
Kinetics of BA degradation by *D. hansenii* H525 as detected by TLC. Cells were incubated at 30 °C in phosphate buffer with 0.25 mM of each amine. One sample contained only 0.25 mM ammonium sulfate and remained non-inoculated. The arrow indicates direction of separation.

The most effective strain *D. hansenii* H525 was studied in more detail. The degradation of phenylethylamine, tyramine, histamine, and ethanolamine was tested with cell suspensions in phosphate buffer. After seven days of incubation, no more bands for phenylethylamine and tyramine could be detected by TLC (Figure 4), indicating their complete degradation by the yeast. The spots for histamine and ethanolamine were still visible, but were of less intensity than in the control. In course of the incubation, a new signal increased, which we interpreted as an ammonium compound. Indeed, it displayed the same R_f_-value as obtained by ammonium sulfate used for comparison. This metabolite can be expected when BAs are degraded by the action of copper amine oxidases (see below).

In the previous experiments only four of the most common amines in wine were added to the medium. Therefore, the substrate range was tested in grape juice in presence of cadaverine, ethanolamine, ethylamine, hexylamine, histamine, isoamylamine, phenylethylamine, putrescine, serotonine, and tryptamine. After 29 days at 30 °C, *D. hansenii* H525 degraded the whole set of amines when added separately or as a mixture to the grape juice as detected by TLC (data not shown). The same was observed for *Yarrowia lipolytica* H446, however this strain degraded tryptamine only when present with other biogenic amines in the mixture.

### 3.3. Degradation of BA by Peroxisomal Enzymes from D. hansenii H525

In order to get more insights in the mechanisms of BA degradation by *D. hansenii* H525, attempts were made to demonstrate and characterize responsible enzyme activities. Due to the presumption that the desired enzyme could be present within yeast cell compartments [19], the cell extract obtained after the French press procedure was subjected to fractionated centrifugation to obtain the peroxisomal fraction. The resulting pellet was washed and treated with the detergent Triton X-100. In this way, peroxisomes and membrane embedded enzymes should go into solution. After ultracentrifugation the resulting supernatant (S3) was concentrated twice by ultrafiltation. After 24 h incubation with extract S3, all four tested amines were significantly reduced in concentration. Histamine and phenylethylamine could no longer be detected by TLC, and tyramine and ethanolamine were present only in traces (not shown).

**Figure 5:**
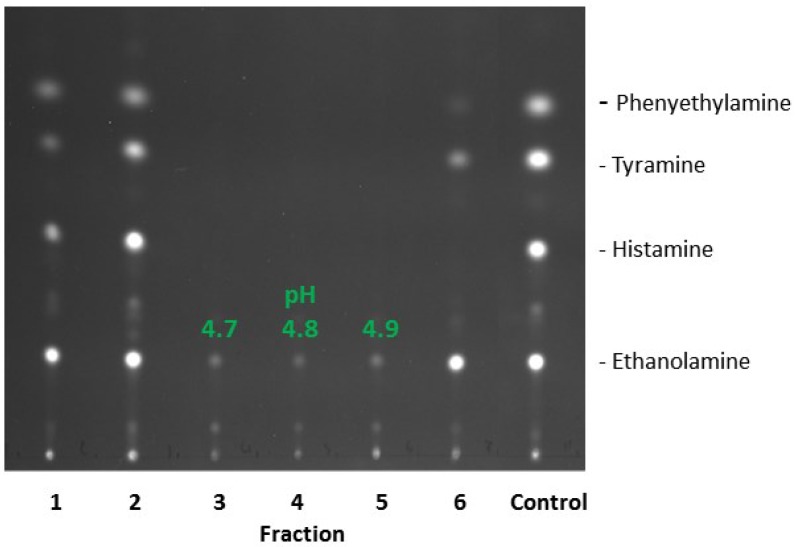
Degradation of BA by a cell-free extract (S3) from *D. hansenii* H525 as detected by TLC. Six fractions of S3 obtained after pIEF were incubated with 0.25 mM BA for 24 h.

For further purification, extract S3 was separated by preparative isoelectric focusing (pIEF). In order to estimate the isoelectric point of the searched protein, extract S3 was previously subjected to analytical isoelectric focusing (aIEF). The majority of the proteins focused in the range of 4.5 to 5.2 (not shown). Corpillo *et al.* [20] characterized an amine oxidase of the yeast *Kluyveromyces marxianus* with a similar pI of 3.9. Based on these data, the ampholyte solution for the pIEF was selected, which was in the acidic pH range (3–5). As shown in Figure 5, fractions 3–5 from pIEF degraded phenylamine, tyramine, and histamine below the detection limit of TLC (0.5 mg/mL), whereas traces of ethanolamine were still visible.

### 3.4. Detection of a Copper Amino Oxidase Gene (CAO) in D. hansenii H525

The degradation of amines can be catalyzed by different classes of amine oxidases. According to the nature of the prosthetic group they can be divided into FAD-dependent (E.C. 1.4.3.4) and copper-containing amine oxidases (CAOs, E.C. 1.4.3.6). The latter have already been isolated and characterized from several yeast species [21].

Therefore, we checked whether a copper-containing amine oxidase gene is present in *D. hansenii* H525. Based on conserved regions in the *CAO* gene, primers Aox2 F and Aox3 R were developed. The 347 bp PCR product was translated into the corresponding amino acids [YIGMRLKGVIHYMDAHFPDRDGEPSTIKNAICIHEEDDGVLFKHSDFRDDFQTTIVTRGTRLIISQIFTASNYEYCLYWIFRQDGTVKLEIRLTGILNTYICAEYEDVGPWGTQ] and compared with sequences in the NCBI Protein Data Base.

The protein was identified with 92% agreement (104/114 AS) as part of a putative copper amine oxidase (Acc.No. XP_461794) from *D. hansenii* CBS 767. These enzymes belong to the class of type 2 or “non-blue” copper proteins [21] and catalyze the deamination of primary amines, giving the respective aldehydes, with an equimolecular consumption of molecular oxygen and production of hydrogen peroxide and ammonia (RCH_2_NH_2_ + H_2_O + O_2_→RCHO + NH_3_ + H_2_O_2_).

## 4. Conclusions

*D. hansenii* is well known for its high osmotolerance. The species has been regularly identified in high salt environments like cheese [22] or high sugar environments like grape must [23]. Strains of *D. hansenii* and *Y. lipolytica* have already been tested for their potential as starter cultures for cheese production [24]. As our isolates H525 and H466 have been recognized as efficient BA degraders they might be appropriate candidates for such purposes. Due to low ethanol tolerance and possible generation of off-flavours the use of *D. hansenii* H525 as starter culture for wine production, e.g., in cofermentions with *S. cerevisiae* appears problematic. However, the application of yeast’s amine oxidase to reduce BA contents in food might be an alternative strategy, which affords further investigations.
